# How, with whom and when: an overview of CD147-mediated regulatory networks influencing matrix metalloproteinase activity

**DOI:** 10.1042/BSR20150256

**Published:** 2016-01-15

**Authors:** G. Daniel Grass, Bryan P. Toole

**Affiliations:** * Department of Radiation Oncology, H. Lee Moffitt Cancer Center and Research Institute, Tampa, FL 33612, U.S.A.; † Department of Regenerative Medicine & Cell Biology, Medical University of South Carolina, Charleston, SC 29425, U.S.A.; ‡ Hollings Cancer Center, Medical University of South Carolina, Charleston, SC 29425, U.S.A.

**Keywords:** basigin, cancer, CD147, EMMPRIN, matrix metalloproteinase, protein oligomerization

## Abstract

Matrix metalloproteinases (MMPs) comprise a family of 23 zinc-dependent enzymes involved in various pathologic and physiologic processes. In cancer, MMPs contribute to processes from tumour initiation to establishment of distant metastases. Complex signalling and protein transport networks regulate MMP synthesis, cell surface presentation and release. Earlier attempts to disrupt MMP activity in patients have proven to be intolerable and with underwhelming clinical efficacy; thus targeting ancillary proteins that regulate MMP activity may be a useful therapeutic approach. Extracellular matrix metalloproteinase inducer (EMMPRIN) was originally characterized as a factor present on lung cancer cells, which stimulated collagenase (MMP-1) production in fibroblasts. Subsequent studies demonstrated that EMMPRIN was identical with several other protein factors, including basigin (Bsg), all of which are now commonly termed CD147. CD147 modulates the synthesis and activity of soluble and membrane-bound [membrane-type MMPs (MT-MMPs)] in various contexts via homophilic/heterophilic cell interactions, vesicular shedding or cell-autonomous processes. CD147 also participates in inflammation, nutrient and drug transporter activity, microbial pathology and developmental processes. Despite the hundreds of manuscripts demonstrating CD147-mediated MMP regulation, the molecular underpinnings governing this process have not been fully elucidated. The present review summarizes our present knowledge of the complex regulatory systems influencing CD147 biology and provides a framework to understand how CD147 may influence MMP activity.

## DISCOVERY, AND GENE AND PROTEIN STRUCTURE OF CD147/EMMPRIN/BASIGIN

Cluster of differentiation 147 (CD147) was initially identified in various species and tissues as the antigens RET-PE2 [1], CE9 [2] and OX-47 [3] in rats, antigen gp42 [4] and basigin [5] in mice, antigen HT7 [6], neurothelin [7] and antigen 5A11 [8] in chickens, and human leucocyte activation-associated antigen M6 [9], blood group Oka antigen [10], hepatoma-associated antigen HAb18G [11] and extracellular matrix metalloproteinase inducer (EMMPRIN) [12] in humans.

In the mid to late 1980s, the Biswas laboratory characterized a factor present on lung carcinoma cell membranes that stimulated MMP-1 production by fibroblasts. By co-culture techniques, they initially discovered a protein with diverse molecular masses that was present on the tumour cell surface and in conditioned media, which functioned as a tumour cell-derived collagenase stimulatory factor, thus named TCSF [13–15]. In addition to MMP-1 stimulation, TCSF was found to promote increased message and protein levels of MMP-2 and MMP-3 and was renamed EMMPRIN to denote a more global role in regulating MMPs [12,16]. It has been demonstrated that the aforementioned proteins are identical and now are commonly termed CD147/emmprin/Bsg, though some investigators still employ earlier nomenclature [9,17,18].

**Figure 1:**
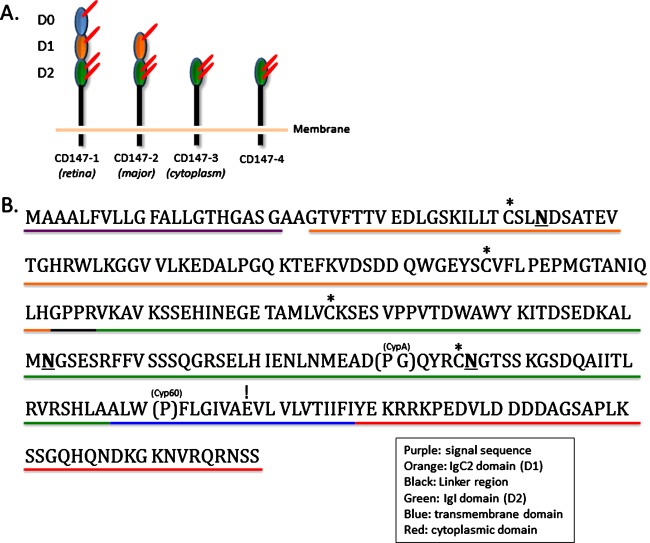
Protein domain structure of CD147 variants (**A**) Extracellular domains identified in each CD147 variant and their expression/localization patterns. D0: retina-specific domain; D1: IgC2 domain; D2: IgI domain. *N*-linked glycosylations are indicated by red lines attached to domains. (**B**) Amino acid sequence of human CD147-2. Each domain of CD147 is specified by the following underline colour: signal sequence (purple), IgC2 domain (orange), linker region (black), IgI domain (green), TM domain (blue) and cytoplasmic domain (red). The linker, TM and cytoplasmic regions are highly conserved but the Ig domains are variable among species. Highly conserved cysteine residues, asparagine *N-*glycosylation sites and TM glutamate residue are indicated by asterisks, underlined bolded letters and exclamation point respectively. Residue-specific binding sites for cyclophilin-A (CypA) and Cyp60 are indicated by parentheses.

CD147, then termed Bsg, was originally cloned from F9 embryonal carcinoma cells as a receptor for *Lotus tetragonolobus* agglutinin, which binds sialyl Lewis X [5]. The human gene, *BSG*, is located on chromosome 19 at p13.3 [19] and contains 10 exons [20,21], whereas in mice *bsg* is localized to chromosome 10 [22–24]. *BSG* encodes four variants through alternative promoters and splicing [20,21], termed CD147/Bsg-1, -2, -3 and -4: a retina-specific variant containing three Ig-like domains (CD147/Bsg-1) [25,26], two variants containing a single Ig-like domain (CD147/Bsg-3 and -4) [20,21] and CD147/Bsg-2, the most abundant and best characterized isoform, which contains two Ig-like domains **(** Figure 1A**)**. Hereafter CD147/Bsg-2 will be referred to as CD147 unless specified otherwise. Sequence analysis demonstrated that CD147 is a single-chain type I transmembrane (TM) protein and a member of the immunoglobulin superfamily (IgSF). The human mRNA transcript encodes a 269 amino acid protein composed of a 21 amino acid signal sequence, a 186 residue-long extracellular portion consisting of two Ig-like domains at the N-terminus, a 21 amino acid TM domain and a 41 residue cytoplasmic domain at the C-terminus [27] **(** Figure 1B**)**.

CD147 has homology to both the MHC II β-chain and Ig variable domain (V); this has led some investigators to speculate that CD147 may be an evolutionary intermediate between a primordial Ig form and MHC II-β chain-like and V domain-containing molecules [3–5,17,28]. In line with this, high resolution crystallography revealed that CD147 contains a N-terminal constant 2-set arrangement (IgC2) domain and a membrane proximal intermediate set (IgI) domain that are organized in a unique manner, distinguishing it from other IgSF proteins [29].

The protein sequence shows varying degrees of conservation across several species, especially in the extracellular domains, but the linker sequence between the Ig-like domains, the cysteine residues, asparagine glycosylation sites, TM domain and cytoplasmic domain demonstrate strong homology [3,6,23,29,30]. Interestingly, the highly conserved regions of CD147 contain distinctive structural characteristics, such as a flexible 5-residue linker domain that has been shown to provide CD147 a great deal of domain mobility, possibly allowing the IgC2 domain to alter its orientation to interact with ligands or adjacent binding partners [29]. The TM domain contains a uniquely-embedded glutamic acid residue as well as a leucine zipper motif [18]. Proteins with these characteristics have been shown to oligomerize into multi-protein complexes and are often involved in cell signalling events, such as immune cell receptor complexes [31]. Proclivity for homo- or hetero-oligomerization may be due to the combination of a polyleucine-rich TM domain with an embedded glutamate residue, which promotes strong interactions between α-helices, possibly via hydrogen bonding [32].

The predicted molecular mass of CD147 is 27–29 kDa, yet many investigators found that CD147 migrates between 31 and 65 kDa with western blotting. This variance has been attributed to differential glycosylation at three conserved asparagine (*N*)-glycosylation sites, Asn^44^, Asn^152^ and Asn^186^ [5,28]. Studies with glycosylation inhibitors and specific lectins revealed that approximately 50% of the mass of CD147 is due to carbohydrate side groups bearing β1,6-branched, polylactosamine-type sugars, fucosylations, Lewis X epitopes and sialylations [33–37]. Furthermore, site-directed mutagenesis at each of the *N*-glycosylation sites demonstrated a relatively equal decrease in the molecular mass, suggesting glycosylation at each attachment site may be equally proportioned [33].

CD147 glycoforms are characterized as low-glycosylated (LG) or high-glycosylated (HG), representing ∼32 kDa and ∼45–65 kDa respectively [33]. LG-CD147 contains high-mannose carbohydrate chains, whereas HG-CD147 contains branched polylactosamine chains that have been processed by Golgi-resident *N*-acetylglucosaminyltransferase V (GnT-V) [33,37]. Though less characterized, GnT-IV may provide the branched core structure on CD147 needed for further GnT-V processing [38].

In further support of a LG- to HG-CD147 precursor–product relationship, a recent study proposed that LG-CD147 is inefficiently processed to HG-CD147 in the endoplasmic reticulum (ER) and that residual unprocessed LG-CD147 is degraded via ER-associated degradation (ERAD) [39], which is independent of SEL1, though requires mannose trimming [40]. In contrast, hepatocellular carcinoma cells exposed to ER stressors, which induce the unfolded protein response, increased CD147 expression on the cell surface, thus avoiding ERAD [41]. Why ER stress increases CD147 surface levels is unclear, but may depend on the vital role of CD147 in chaperoning nutrient transporters to the plasma membrane or activating survival pathways by diverse protein–protein interactions. Site-directed mutagenesis revealed that *N*-glycosylation at Asn^152^ regulates protein quality control, whereas disruption of other *N*-glycosylation sites on CD147 had no effect on surface localization [37]. Thus, some pools of LG-CD147 may be degraded whereas other efficiently processed LG-CD147 pools are trafficked to the cell surface to participate in diverse protein–protein interactions, though to a lesser extent compared with HG-CD147 [33,42]. Others have demonstrated that the conversion of LG- to HG-CD147 is regulated by interactions with caveolin-1 [43], monocarboxylate transporters (MCTs) [44] or cyclophilin 60 (Cyp60) [45,46]. It is clear that CD147 is heterogeneously glycosylated across many tissue and cell types [47] and even shows variations in glycosylation in cell lines derived from the same tissue of origin [36]. Unraveling the intricacies of CD147 glycosylation is just beginning and the authors encourage readers to read a recent review by Bai et al. [48] for further details.

## DIVERSE EXPRESSION PATTERNS AND FUNCTIONS OF CD147 IN PHYSIOLOGIC AND PATHOLOGIC CONTEXTS

CD147 is expressed in many epithelial, neuronal, lymphoid and myeloid cell types [3,9,17,47,49], though as various glycoforms. Tissue arrays of CD147 expression in normal and cancer tissues demonstrate that CD147 is mainly restricted to normal tissues of the reproductive tract, brain, eye and muscle, whereas the majority of malignant cancers have elevated expression [36,50]. It is clear that CD147 is overexpressed in a variety of cancers [51,52] and is also widely expressed and diversely functional during developmental processes, wound healing, nutrient transport, inflammation, atherosclerosis, arthritis and microbial pathologies, as reviewed elsewhere [53–63].

Two of the earliest credited functions of CD147 include MMP induction [14] and cell recognition during neuronal-glial patterning and aggregation [8]. Knockout studies in mice revealed that the majority of CD147-null (*bsg^−/−^)* mice die around the time of initial blastocyst implantation, though different unknown modifier regions surrounding the CD147 gene may attenuate this death rate [64,65]. In the rare event that an embryo successfully implants, the offspring are small and usually die before one month due to difficulty in breathing secondary to interstitial pneumonia. Surviving males are sterile due to defects in spermatogenesis [66,67] and null females have problems with fertilization [65,68]. In addition, CD147-null mice display abnormalities in spatial learning, memory and sensory perception to painful stimuli and noxious odours [69,70], in early retinal function leading to blindness [71–73], in tooth development [74] and in wound responses and lymphocyte reactions [55,75].

Evaluation of CD147 function in other model systems has highlighted additional biological roles. For instance, up-regulation of CD147 in High Five insect cells induces drastic changes in the organizational structure of the cytoskeleton, which is independent of cell–cell contact or exposure to conditioned media [76]. In *Drosophila*, depletion of CD147 causes lethality and specific knockdown in the eye leads to misplaced sub-cellular organelles in photoreceptor cells [76]. Furthermore, CD147-depleted flies have misplaced glial cell–photoreceptor interactions and altered synaptic vesicle release [77]; these phenotypes may be secondary to a conserved YEKRRK sequence in the cytoplasmic tail [78]. Thus, data from the knockout models suggest CD147 has a multitude of functions including regulation of cytoskeletal remodelling, assembly of cell–cell interaction modules and sub-cellular vesicle distribution that span an array of biologic functions.

## CELLULAR LOCALIZATION AND VESICULAR TRAFFICKING OF CD147

Although tissue patterns of CD147 expression have been widely studied, the patterns of expression on a cellular level have not been described until recently. CD147 was originally identified on the cell surface with a tendency to localize at cell–cell interfaces [7,14,79]. As imaging techniques have improved, it is evident that CD147 also resides in sub-cellular compartments and in vesicles released from the cell. A novel study by Eyster et al. [80] demonstrated that an Arf6-GTPase mutant expressed in HeLa cells led to enlarged vacuolar-type structures enriched with bulk membrane clathrin-independent endocytic (CIE) cargo, which included CD147, CD44 and CD98. In this study CD147, CD44 and CD98 rapidly joined recycling tubular endosomes via the juxtanuclear endocytic recycling compartment. Additionally, paired acidic residues in the cytoplasmic tail of CD147 interact with Hook1, a microtubule-binding protein, which cooperates with Rab22a in sorting CD147 away from early endosomal antigen 1 (EEA1)-associated vesicles and subsequent transfer to late endosomes for lysosomal degradation [81]; furthermore, cell surface expression of CD147 was minimally affected by up-regulation of membrane-associated RING-CH E3 ubiquitin ligases, which reduced surface expression of other CIE cargo members [82]. Rapid intracellular transit of CD147, in contrast with other CIE cargo, may be due to the dynamic processes this protein partakes in, such as nutrient flux across the cell membrane and ECM remodelling. In contrast with these findings, others have shown that CD147 requires clathrin-dependent sorting to localize to the basolateral membrane [83,84] and our laboratory showed that pools of CD147 associate in EEA1-containing vesicles in breast epithelial cells [85]. It should be noted that the majority of work describing CD147 trafficking via CIE has been performed in HeLa cells; thus it is difficult to generalize this process to other cell types.

Additionally, CD147 can associate with Annexin II (AnxA2), a multifunctional protein that regulates cytoskeleton organization, lipid raft dynamics and endo/exocytic trafficking events [86]. This interaction occurs via the extracellular portion of CD147 and may influence AnxA2 phosphorylation, cell motility and release of CD147 from cells [87–89]. A growing amount of evidence also suggests that CD147–CD147 interactions may lead to internalization and binding of CD147-3 in uncharacterized cytoplasmic vesicles [21,90], though the consequences of these events are still unknown. Thus, the mechanisms of CD147 sub-cellular sorting are probably dependent on the tissue type, microenvironmental cues and the dynamic cooperation of diverse endocytic machinery [91].

In addition to plasma membrane and sub-cellular vesicle localization, CD147 has also been identified in the extracellular milieu. The initial characterization of CD147 in cancer cells demonstrated a proportion of CD147 in tumour-conditioned media [14] and subsequent studies have shown that CD147 is released from the cell surface in a full length soluble form [92] or a 22-kDa proteolytic cleavage product not associated with vesicles [93]. Others have provided evidence that CD147 is released in microvesicles or exosomes, which can interact with distant cells [94,95]. CD147-associated exosomes have been identified in malignant ascites from ovarian cancer patients [96] and in bladder cancer [97], and recent evidence suggests that CD147-containing extracellular vesicles can be extracted from cancer patient sera as a biomarker to monitor response to therapy [98]. Clearly, characterization of the released pools of CD147 and the effects of internalized CD147–CD147 complexes are emerging fields of discovery.

## REGULATION OF CD147 EXPRESSION

**Figure 2:**
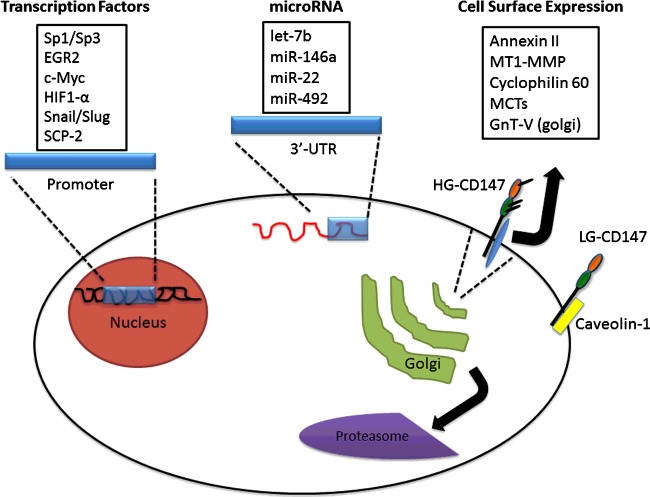
Regulators of CD147 transcription, translation and plasma membrane expression Various TFs cooperate to regulate CD147 transcription in different biological contexts, which may influence transcription initiation at different alternative promoters or influence unknown splicing machinery. Following transcription, CD147 message levels may be further regulated by multiple miRNAs. Prior evidence suggests that newly synthesized CD147 is inefficiently processed in the ER and Golgi and the majority is degraded by the proteasome. It is hypothesized that constitutive production of CD147 ensures adequate protein pools to respond to dynamic changes in cellular needs [39]. Appropriately processed CD147 is then trafficked to the cell surface to assist in MMP activity, nutrient transport and cell signalling pathways. Each of these processes is regulated by a diverse cohort of proteins (see text for details), though how these proteins cooperate in regulating CD147 surface expression is unknown.

Complex regulatory circuits govern transcription, translation and cell surface presentation of CD147 (Figure 2), and each of these mechanisms is likely to be cell type dependent. Initial characterization of the *CD147* promoter region demonstrated a CpG island (CGI)-rich sequence with a TATA box [24,99]. Over the years consensus binding sites for multiple transcription factors (TFs), including specificity protein 1 (Sp1), Sp3, early growth response protein 2 (EGR2), epithelial–mesenchymal transition (EMT)-associated factors (e.g. Snail and Slug), a transcriptionally active fragment of sterol carrier protein 2 (SCP-2) and hypoxia-inducible factor-1α (HIF-1α), have been identified [24,100–104]. The majority of evidence suggests that Sp1 is one of the main TFs regulating CD147 expression [104,105] and a recent paper demonstrated cooperative interactions between c-Myc and Sp1 in the *CD147* promoter region [106]. Due to the fact that Sp1 binds to CpG motifs present in CGIs, which can be epigenetically modified by methylation, Sp1-mediated transcriptional initiation may be altered by methylated CpG motifs in promoters [107]. In support of this, the *CD147* promoter was found to be hypomethylated in cancer tissue compared with normal tissue, resulting in increased Sp1 binding and consequently increased CD147 expression [108].

Control of CD147 expression by *cis-*regulatory elements and sequence variations/polymorphisms has also been described. Seed regions of let-7, a tumour-suppressive non-protein coding miRNA family, were shown to have complementary sequence to the 3′-UTR of CD147, with let-7b being the most complementary [109,110]. Other miRNAs found to modulate CD147 expression include *miR-146a* [111,112] and *miR-22*, with the latter being governed by a complex regulatory loop involving c-Myc and Sp1 [106]. Additionally, *miR-492* decreases CD147 expression, though the efficiency of suppression appears to be dependent on a single nt polymorphism (SNP) located at the fourth nt complementary to the *miR-492* seed region [113]. It is thought that ∼2–6 contiguous base pairs in the seed region of miRNAs are needed for effective miRNA–mRNA duplex formation and target suppression [114]; hence the SNP in the *miR-492* binding site may alter affinity to the CD147 3′-UTR resulting in differential suppression of CD147.

Many studies have identified factors upstream of the CD147 transcription initiators and repressors mentioned above. Some of the earliest studies identified an increase in CD147 expression following lymphocyte activation by granulocyte macrophage colony-stimulating factor, concanavalin-A or phytohaemagglutinin [9,115]. Additionally, endothelial cells that invade co-cultured brain tissue demonstrated increased CD147 expression [116]. These earlier studies set the premise that CD147 is an inducible molecule in both physiological and pathological contexts.

Over the years a litany of soluble mediators, such as growth factors, cytokines and hormones, has been shown to regulate CD147 transcription and translation. In breast cancer cells, epidermal growth factor (EGF) and amphiregulin induced the expression of CD147 via an EGF receptor (EGFR) pathway [117,118] and transforming growth factor β (TGF-β) increased CD147 expression in a phosphatidylinositol 3-kinase (PI3K)-Akt-dependent manner in hepatocytes [102]. Since CD147 is known to participate in inflammation, ischemic injury, atherosclerosis, rheumatoid arthritis and tissue repair [18,55], it is no surprise that it is regulated by various inflammatory mediators, such as tumour necrosis factor α (TNF-α) [119], interleukins [120], receptor activator of nuclear factor κ-light chain-enhancer of activated B cells (NF-κβ) ligand (RANKL) [121,122] and prostaglandins [123]. Sex hormones, such as progesterone and oestrogen, have been shown to modulate CD147 expression as well [124–126]. Studies with oestrogen receptor-α or -β null mice revealed that CD147 is regulated by oestrogen receptor-α in select tissues, whereas in others it is independent of oestrogen signalling and regulation occurred at the translation level [127]. Other hormones such as thyroid-stimulating hormone (TSH) [128,129] and angiotensin II [130,131] peptides, which signal through G-protein-coupled receptors, have been shown to regulate CD147 expression. Numerous upstream pathways have been implicated in CD147 expression, which are beyond the scope of this manuscript, but are discussed further in a recent review [51]. It is easy to appreciate that diverse signalling mechanisms influence CD147 expression in a variety of tissues.

In addition to the array of signalling pathways, CD147 expression and cellular localization are influenced by interactions with itself and other proteins. CD147 may self-regulate its own expression in an autocrine manner [90,132] possibly via MT1-MMP-dependent cleavage of surface bound CD147 [93]. Notably, treatment of cells with soluble CD147 results in concentration and time-dependent increases in CD147 transcription and surface expression [90]; hence differing baseline levels of CD147 expression in interacting cells may influence biological outcomes.

The best characterized binding partners of CD147 are MCTs, a family of transporters involved in lactate, pyruvate and ketone flux across the plasma membrane [60]. MCTs tightly associate with CD147 and influence post-translational processing in the Golgi as well as localization to the plasma membrane [44,133]. In the majority of scenarios, both proteins are co-dependent on each other for appropriate surface expression, though this was recently shown to be not always the case [134].

Another protein class that regulates CD147 cell surface expression is the cyclosporine A (CspA)-sensitive cyclophilin protein family. Cyclophilins contain peptidyl-prolyl *cis–trans* isomerase activity, are known to regulate protein trafficking, and act as intercellular mediators during inflammation [59]. Prior studies found that CD147 cell surface expression was decreased by treatment with the immunosuppressive drug CspA [46] and that proline 211, located near the CD147 TM domain, facilitates interaction with Cyp60, which may be involved in escorting CD147 to the cell surface [45].

It is apparent that CD147 is regulated by complex signalling networks in different physiological, pathological and tissue-specific contexts. The regulation of CD147 expression by various TFs, soluble mediators and binding partners underscores the complexity of CD147 function.

## POTENTIAL MECHANISMS REGULATING CD147-MEDIATED MMP ACTIVITY

Since the discovery of CD147 approximately 30 years ago, many investigators have demonstrated that CD147 is a major inducer of proteases in an array of non-tumorigenic and tumorigenic cell types; the authors encourage readers to see an exhaustive list of malignancies that have been evaluated for relationships between CD147 and MMPs [51]. To date, CD147 has been shown to mediate expression and activity of soluble and MT-MMPs, including MMP-1, MMP-2, MMP-3, MMP-9, MT1-MMP and MT2-MMP [18,27,50]. It should be noted that the compilation of published literature evaluating CD147-mediated MMP activity has been derived from experiments evaluating MMP message, protein and enzymatic activity in various models, though not always on a consistent basis.

CD147 increases MMP production in fibroblasts, endothelial cells, macrophages, tumour cells and non-immortalized epithelial cells [135]. In addition to MMP induction, CD147 also participates in the activation of the urokinase-type plasminogen activator system in breast cancer, oral squamous cancer, trophoblasts and endothelial cells [136–138], as well as a disintegrin and metalloproteinase with thrombospondin motifs (ADAMTS)-1 and ADAMTS-9 production in cells infected with Kaposi's Sarcoma-associated herpes virus [139]. Even though CD147 has been shown to regulate proteases in a variety of scenarios, the specific mechanisms regulating this process remain ambiguous.

**Figure 3:**
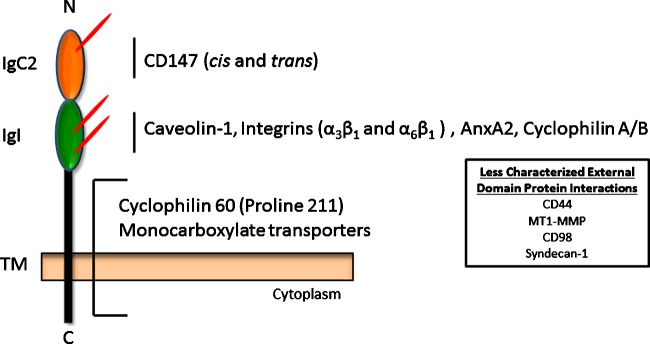
CD147 protein–protein interactions and interacting domains Protein–protein interactions have been localized to specific CD147 domains. The majority of evidence indicates that the N-terminal (Domain 1: IgC2) domain is where homophilic interactions occur and influence MMP activity. The membrane proximal (Domain 2: IgI domain) has been implicated in CD147 associations with caveolin-1, integrins and AnxA2. Additionally, cyclophilins are thought to influence inflammatory signalling pathways via this domain [59]. The TM region of CD147 contains a leucine zipper motif and a unique glutamate residue; these characteristics are thought to increase the propensity of diverse heterophilic CD147–protein interactions, some of which the exact interacting domain on CD147 is unknown. No signalling motifs have been identified in the cytoplasmic tail and the exact function of this domain is unknown. CD147 *N*-glycosylation probably has some role in these protein–protein interactions and its influence is probably dependent on cell type, extracellular cues and available nutrient substrates for glycan synthesis.

**Figure 4:**
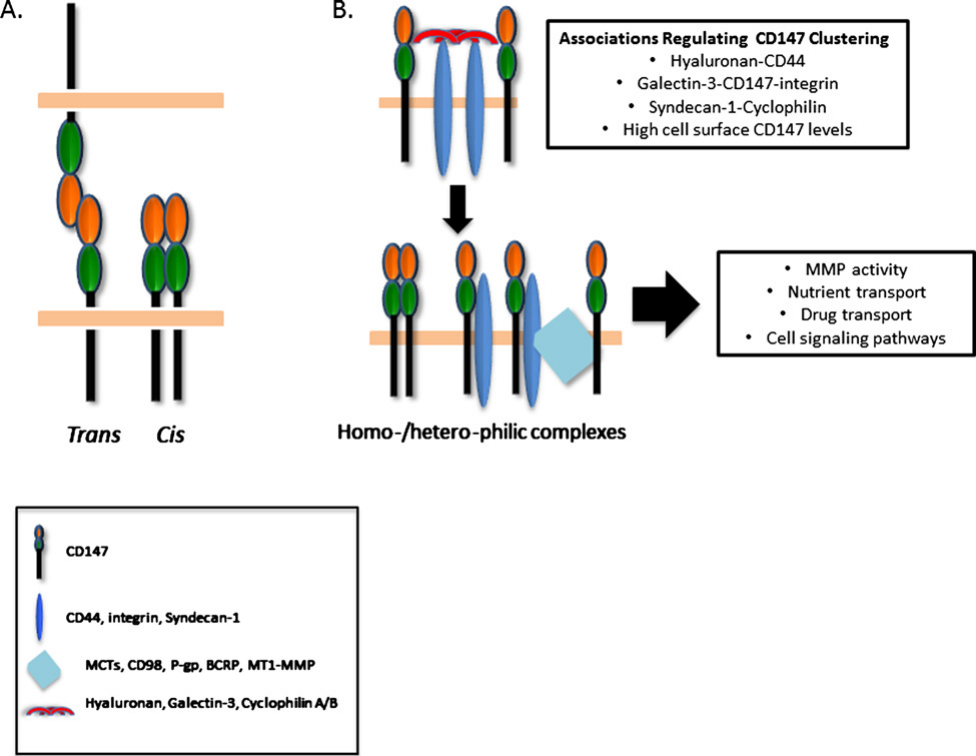
Homophilic/heterophilic interactions between CD147 and other proteins regulate pleiotropic biology of CD147 (**A**) CD147 can self-associate in a *cis* or *trans* fashion between homophilic and heterophilic cell types; depiction of *N*-glycosylation was omitted for simplicity. (**B**) Various factors influence the propensity of CD147 clustering and multicomponent complex formation. Hyaluronan–CD44 interactions influence various components of tumour biology by cross-linking CD44 molecules, which can lead to higher order oligomerization of CD147-containing complexes; these complexes have been shown to regulate MMP activity, nutrient (MCTs) and drug [P-glycoprotein (P-gp), breast cancer resistance protein (BCRP)] transport across cell membranes as well as various cell signalling pathways [135]. Similar to the stabilizing pericellular hyaluronan–CD44 interactions, the carbohydrate-binding domain of Gal-3 is thought to interact with glycans on CD147 and induce clustering with integrins, which can regulate MMP activity. Cyclophilins can also interact with the external domains of CD147, possibly via Syndecan-1 [184]. These interactions can induce CD147 clustering and activation of downstream inflammatory pathways. Greater proportions of CD147 on the cell surface will lead to more CD147-containing complexes and as expected enhanced activity of CD147-dependent functions.

Overviewed above, CD147 expression and cell surface localization is influenced by a diverse set of cues, which in some cases are tissue/cell-type specific. As far as the authors are aware, there has been no report describing intrinsic signalling motifs in CD147. Thus, the pleiotropic biology of CD147 is probably due to a combination of glycosylation status, homo-dimerization/oligomerization and heterophilic protein–protein interactions (Figures 3 and 4), each of which probably orchestrates a facet of MMP regulation.

### Glycosylation

The contribution of *N*-glycosylation to CD147-mediated MMP activity is controversial. The majority of studies describe CD147 as an *N*-linked glycosylated protein, with the exception of one study in chicken retinal tissues, which described both *N*-glycan and *O*-glycan attachments [140]. Prior experiments demonstrated CD147 cDNA expressed in *E. coli* resulted in a non-glycosylated form approximately 29 kDa, which had no MMP-inducing activity when added to fibroblasts. These studies also suggested that recombinant (rb) LG-CD147 (30–45 kDa) was unable to induce MMP synthesis [141]. Subsequent studies demonstrated that deglycosylated endogenous CD147 had no MMP-inducing activity and attenuated the ability of HG-CD147 to induce MMPs [142]. A recent study comparing synthesized CD147 in two expression systems found that only the glycosylated CD147 was able to induce MMP-2 in fibroblasts efficiently [143]. This compilation of data strongly suggests that glycosylation was mandatory for CD147-mediated MMP induction.

Conversely, others have demonstrated that non-glycosylated CD147 [21,144] or a peptide corresponding to the IgC2 domain with a single *N*-acetylglucosamine (GlcNAc) or chitobiose unit [145] were capable of stimulating MMPs. We recently found that up-regulation of CD147 expression in non-transformed breast epithelial cells, which specifically resulted in a higher proportion of the 38 kDa compared with 52–58 kDa glycoform, led to similar induction of MMP activity as the previously mentioned effects of HG-CD147 [85]. Hence, it appears that both LG- and HG-CD147 contribute to MMP activity, though the efficiency of this induction may be more pronounced with HG-CD147 glycoforms [37].

### Homo-dimerization/oligomerization

CD147 was first found to homo-oligomerize in avian tissues, in which the authors proposed that CD147 can potentially interact in *cis* and *trans* fashion forming a macromolecular complex [146]. Later findings suggested CD147 forms homo-oligomers only in a *cis-*like manner and that the IgC2 domain was sufficient for this dimerization whereas *N*-glycosylation appeared to play no role [147]. In contrast others have provided evidence that CD147 Ig domains do not dimerize in solution [90].

Since tumour cell-associated CD147 and soluble CD147 have been reported to induce MMP synthesis in neighbouring cells, this implies that a counter-receptor may participate in *trans*-interactions between these cells [18]. Studies employing an immobilized CD147 fusion protein determined that CD147 can act as a receptor for itself, similar to other IgSF members, and that this association was dependent on the IgC2 domain. Additionally, MMP induction was inhibited by an antibody that specifically bound multimerized CD147, suggesting CD147 oligomerization facilitates MMP induction [142]. Furthermore, cross-linking between the Ig-like domains of rbCD147 and fibroblasts followed by MALDI-MS/MS sequencing identified CD147 as a receptor for rbCD147 [21] and crystallographic approaches identified that Ig-like domains in CD147 dimerize in both *cis* and *trans* fashion [29] possibly through β-strand domain swapping [148]. Also, systematic mutational analysis of the ectodomain of CD147 revealed that various mutations in the Ig-like domains inhibited dimerization/oligomerization and subsequently MMP induction [149]. Thus, in certain contexts CD147 can self-associate, but there may be differences in this process when comparing soluble with membrane bound CD147.

Oligomerization of surface proteins can occur by extracellular ligands directly interacting with external domains of the target protein or by binding to neighbouring partners that indirectly influence clustering of the target protein. Galectins are a large family of lectins that bind β-galactoside moieties and perform diverse functions in biology [150,151]. Within this family, galectin-3 (Gal-3) uniquely has a GlcNAc-binding C-terminal domain and an N-terminal domain that facilitates self-association, thus functioning as an external cross-linking protein lattice [152]. In this respect, proteomic cell surface analysis identified that Gal-3 promotes integrin β1 and CD147 clustering; the latter interaction is likely secondary to cross-linking of branched glycans on CD147 [153]. Gal-3–CD147 interactions were also found to redistribute CD147 to cell–cell contact points, which resulted in MMP-9-dependent loss of occludin and cell–cell attachment [154]. Analogous to the direct extracellular interactions of Gal-3 and CD147, binding of hyaluronan, a large extracellular polysaccharide, to CD44 can also induce cell surface clustering and multicomponent complex formation, which can lead to CD147 oligomerization [135].

Other less characterized mechanisms of CD147-mediated MMP activity have been proposed. There is some evidence that the membrane-proximal IgI domain of CD147 may also regulate MMP activity, though this needs further validation [143]. It should also be noted that CD147-3 binding to the IgI domain of CD147 may attenuate induction of MMP synthesis, possibly functioning as a dominant negative in certain contexts [20]. Additionally, CD147–CD147 interactions and the biologic effect they promote may depend on receptor saturation and propensity to cross-link [90]. Overall, there is strong evidence that in specific contexts CD147 self-associates either in a homo- or heterotypic manner, which propagates signals that influence MMP activity.

### Heterophilic protein interactions

The protein structure and associated binding characteristics of CD147 imply a role in modulating protein–protein interactions. As described previously, CD147 contains a unique glutamate residue as well as a leucine zipper motif in its TM domain [155]; both of these characteristics are associated with multi-protein complex assembly and receptor dimerization [31,32,156]. Furthermore, the IgC2 domain may also interact with IgI/V-like domains on other IgSF proteins [29] and CD147 may associate with proteins with attached oligomannose moieties [157]. In addition to these characteristics, differential glycoforms of CD147 also provide prodigious advantages to participate in diverse protein–protein interactions.

Inasmuch, various binding partners have been described to interact with CD147 in regulating MMP activity. For instance, CD147 associates with caveolin-1 mostly on the cell surface via its membrane proximal IgI domain and this complex attenuates CD147 self-aggregation [43]. Further evaluation of this association revealed that LG-CD147 preferentially associated with caveolin-1 and this interaction inhibited CD147-induced MMP induction [33]. These investigators proposed two divergent pathways: (1) LG-CD147 associates with caveolin-1, which prevents further glycan processing by GnT-V, and is escorted to the cell surface forming caveolin-1-LG-CD147 complexes not involved in MMP activity or (2) LG-CD147 is modified by GnT-V, forming HG-CD147, which does not bind caveolin-1 and is subsequently trafficked to the cell surface to form multimers that increase MMP activity. Hence, caveolin-1 may influence MMP production by diminishing cell-surface clustering and further complex glycosylations of CD147, though these processes appear to be independent of each other [33,43]. Conversely, studies evaluating MMP synthesis in a lung injury model and in hepatocellular carcinoma progression suggest that an inverse relationship exists between caveolin-1 and CD147 and actually increases the proportion of HG-CD147 [158,159].

As mentioned previously, AnxA2 can interact with the extracellular domains of CD147 and influence cancer cell migration and invasion by regulating MMP activity [88,89]. This effect may be secondary to AnxA2 regulation of CD147-associated microvesicle release, which can be a means of communication between tumour and stromal cells [87].

CD147 also forms complexes with α3β1 and α6β1 integrins at cell–cell contacts [160]. Asp^179^ in the IgI domain of CD147 can interact with the metal ion-dependent adhesion site in the βA domain of the integrin β1 subunit, which regulates MMP activity [161]. CD147–integrin interactions promote cancer invasiveness by inducing MMP synthesis via a focal adhesion kinase (FAK)-PI3K signalling pathway [162–164]. β1 integrin also forms a heterocomplex with CD147 and CD98, in which failure of CD147 to associate with the latter results in decreased MMP activity [165]. In oral cancer cells CD147 interacts with β6 integrin and this may cooperate with Fyn, a Src family kinase, in modulating MT1-MMP activity [166]. As mentioned, CD147 also interacts with caveolin-1 via the membrane-proximal IgI domain [43], but whether competing interactions between integrins, caveolin-1 or RGD-containing ligands influence CD147-mediated MMP activity is unknown. From this data it can be reasoned that diverse CD147–integrin interactions are present in cancer cells and regulate multiple processes.

Previously we demonstrated that increased expression of CD147 was associated with invadopodia activity, which was MT1-MMP dependent [85]; others have also found that CD147 regulates MT1-MMP synthesis and cell surface expression [90,167–169]. Of note, elevated MT1-MMP expression was observed after up-regulation of CD147 in our study, though the majority of MT1-MMP remained in the cytoplasm and the proportion that was trafficked to the cell surface probably resided in CD147-enriched lipid rafts. Also, distinct pools of CD147 were found to associate with MT1-MMP in vesicular compartments, which were different from the CD147–MT1-MMP complexes identified in active invadopodia, possibly representing subsets in transit to or from the cell surface [85].

MT1-MMP regulation is very stringent, as the increased presence of a highly proteolytic enzyme on the cell surface could be detrimental to cells not needing to cleave matrix substrates or cell surface factors that promote cell motility. Similar to other MMPs, MT1-MMP is synthesized as a zymogen that needs processing to the active form and in the case of MT1-MMP this occurs via a furin-mediated cleavage event in the *trans*-Golgi network during trafficking to the cell surface. MT1-MMP may then be endocytosed and recycled back to the cell surface or sent for degradation, thus active MT1-MMP is the predominant form localized on the cell surface [170]. Some evidence suggests that CD147 associates with both pro and active forms of MT1-MMP, which may suggest that CD147 participates in the regulatory network of MT1-MMP trafficking and processing [93,171].

As above, CD147 also interacts with CD44, a major receptor for hyaluronan [172], and these formed complexes have been shown to regulate diverse aberrant cancer-promoting signalling pathways and promote stabilization of plasma membrane localized metabolic/drug transporter complexes [135,173–177]. Additionally, we observed that CD147-mediated MT1-MMP-dependent invadopodia activity was partially dependent on CD44–hyaluronan interactions that facilitated CD44–EGFR–CD147 heterocomplex formation in lipid rafts, which resulted in Ras-ERK (extracellular signal regulated kinase) signalling. Furthermore, enrichment of cells with high endogenous surface expression of CD147 identified distinct cell populations with increased potential to invade as well as enhanced mitogen-activated protein kinase (MAPK) signalling [42].

## CONCLUSIONS AND PERSPECTIVES

MMP regulation is governed by complex and redundant mechanisms in physiologic and pathologic cellular processes. CD147 has emerged as a dominant modulator of MMP activity from transcription to cell surface presentation. As CD147 has no identified intrinsic signalling motif in its protein structure, the regulatory effects of CD147 are probably dependent on characteristic TM and glycosylation features that facilitate higher order oligomerization with self or other binding partners. Additionally, pools of released CD147 may interact with counter receptors on adjacent or distant cells and influence MMP activity. Multiple heteromeric CD147-containing complexes have been identified and in certain biologic contexts these can influence MMP activity and other CD147-related processes (Figure 4). Due to the ‘promiscuity’ of CD147 interactions on the cell surface, downstream signalling events may depend on the quantities of CD147 and known binding partners present on the plasma membrane. It can be speculated that cells with high CD147 surface expression may have a higher probability to interact with various binding partners and thus influence MMP activity in diverse manners. In this regard, it is clear that different functional phenotypes are identified when comparing cells with high and low surface expression of CD147 [42,178,179]. The possibility that tumour cells with high levels of cell surface CD147 exhibit cancer stem-like cell properties [178], especially with respect to the role of CD147-induced MMP activities in invasiveness and metastasis, is an important area of future investigation.

Despite numerous manuscripts identifying CD147-mediated MMP regulatory networks, there does not appear to be a unifying mechanism explaining this phenomenon. In fact, some investigators have demonstrated CD147 independence in MMP-dependent processes such as mammary gland development [180] and embryo implantation [181]. Recently, the involvement of CD147 in MMP regulation has been called into question by CD147 knockout studies demonstrating no difference in soluble and MT-MMPs levels/activity; these investigators suggest that all evidence to date implicating CD147 in MMP regulation is indirect and that the main role of CD147 is in the regulation of lactate transport via MCTs [182]. It will be important to resolve the apparent contradiction between these studies and the large body of data pointing towards important roles for CD147 in regulation of MMP expression.

CD147 biology is complex and various stimuli from soluble mediators to pericellular matrix scaffolds influence CD147-dependent processes. Additionally, evidence indicates that the surface expression level of CD147, not total protein amount, is a major predictor of CD147-mediated effects [42,178,183]. Though CD147 was originally identified as a tumour-derived MMP inducer, it is evident that CD147 has pleiotropic functions, which cannot be encompassed by the acronym EMMPRIN [135]. Therefore, the authors propose employing CD147 in future studies.

## Abbreviations

AnxA2: Annexin II
Bsg: basigin
CD147: cluster of differentiation 147
CGI: CpG island
CIE: clathrin-independent endocytosis
CspA: cyclosporine A
Cyp60: cyclophilin 60
EEA1: early endosomal antigen 1
EGF: epidermal growth factor
EGFR: EGF receptor
EGR2: early growth response protein 2
EMMPRIN: extracellular matrix metalloproteinase inducer
ER: endoplasmic reticulum
ERAD: endoplasmic reticulum-associated degradation
Gal-3: galectin-3
GlcNAc: *N*-acetylglucosamine
GnT: *N*-acetylglucosaminyltransferase
HG: high-glycosylated
HIF-1α: hypoxia-inducible factor-1α
IgSF: immunoglobulin superfamily
LG: low-glycosylated
MCT: monocarboxylate transporter
MMP: matrix metalloproteinase
MT-MMP: membrane-type matrix metalloproteinase
PI3K: phosphatidylinositol 3-kinase
rb: recombinant
SNP: single nt polymorphism
Sp1: specificity protein 1
TCSF: tumour cell-derived collagenase stimulatory factor
TF: transcription factor
TM: transmembrane
